# Collectanea Medica, Consisting of Anecdotes, Facts, Extracts, Illustrations, Queries, Suggestions, &c.

**Published:** 1818-01

**Authors:** 


### COLLECTANEA MEDICA,

CONSISTING OF

### ANECDOTES, FACTS, EXTRACTS, ILLUSTRATIONS, QUERIES, SUGGESTIONS, &c.

RELATING TO THE

*History or the Art of Medicine, and the Auxiliary Sciences.*

Quicquid agunt medici, nostri farrago libelli.

THE following Letters, extracted from the several Newspapers in which they have appeared, have been thought worth the registering, to show the various opinions of the medical men of the day on the health of the town.

FEVER INSTITUTION.

*To the Editor of the Morning Chronicle.*—SIR,—In a letter which appeared in your paper two or three days ago, a physician residing at the west end of the town, and obviously destitute of the means of obtaining accurate information, has ventured to assure the public that London is not at present more infected with contagious fever than usual; and that, in fact, such a fever always prevails in the metropolis to a considerable extent. Holding the office of Physician to the Fever Institution, I deem it incumbent on me to endeavour to correct the impression which such an erroneous statement is calculated to produce on the public mind, conceiving that, however desirable it may be to allay groundless and exaggerated alarms, an incautious supposition of security may be equally prejudicial to the public interest. That contagious fever has been rarely seen in London during the last fourteen years, the records of the Fever Institution most clearly demonstrate; to say nothing of the testimony of all the physicians to hospitals and dispensaries, who will concur in the assertion of this fact. The House of Recovery, which was instituted during an epidemic in 1801 (the consequence of two preceding years of scarcity), and was opened in the beginning of 1802, received in the first year 164 patients; in the second, 170; in the third, only 80; and since that time the number admitted annually has been generally much less, being (on the average of the last ten years) but 64; for the whole number of patients during that period amounts but to 644. In the year 1809, indeed, only 29 persons were admitted; and, during all these years, the house has frequently remained empty for several weeks, although open at all times for the immediate reception of every poor person attacked with fever, on application to the physician. It cannot be said, therefore, that contagious fever is usually prevalent in London. But a reference to the same records will shew that the case is very different at present. From the 25th of April last, to this day (Oct. 20th), a period short of six months, 321 patients, affected with typhus fever, have been admitted into the House of Recovery, in the Pancras-road; that is, *half as many as were received during the preceding ten years*; and, of this number, 236 during the last three months,—namely, since the 25th of July; and in the following proportions, from which the progress of the epidemic will be manifest:—from July 25th to August 25th, the admissions were 63; from that day to September 25th, 81; and from the last mentioned day to the present (Oct. 20th), 92. The House is now completely full, containing 64 patients, who occupy also the wards appropriated to scarlet fever, which fortunately does not prevail.

It is somewhat singular, that, at the moment when Dr. Brine takes upon himself to assure the public that there is no unusual quantity of contagious fever in London, that Institution was for the first time obliged to refuse admission to many applicants for want of room: for, on Saturday last, the day on which his letter appeared in several papers, no less than 25 patients claimed admission, only 16 of whom could be received; and, in consequence of the continued daily applications, the Institution still remains under the painful necessity of excluding a part of them.

It is evident, therefore, that there is an epidemic fever at present spreading among the poor of the metropolis, and that in the proportion of about fifteen to one above the ordinary average, comparing the admissions of last quarter (236) with the average quarter for ten years back (16). The evidence of the increase of fever, and in this proportion, does not rest, however, entirely upon the authority of the records of the House of Recovery; for I have the permission of my friend Dr. Marcet, physician to Guy's Hospital, to state that, among the admissions to that Hospital during six weeks, from September 8th to October 15th, last year (1816), there were but three cases of fever: whereas, within the same period this year, there were 47; which is rather more than fifteen to one.

This fever has occurred principally in the close alleys in the parishes of Shadwell, Whitechapel, Shoreditch, St. Luke, St. Sepulchre, and St. Andrew, Saffron-hill; and also those of St. Saviour and St. George, in the Borough: but it has been, and continues to be, particularly rife in Whitechapel, and the alleys about Cow-cross and Saffron-hill, near Smithfield. It has arisen unquestionably from the general want of sustenance among the poor, during the late season of scarcity and deficient employment. It is doubtless contagious, but chiefly, almost exclusively so, in the close and crowded habitations of the indigent; and the public may rest assured that the contagion cannot be communicated in the open air, nor in well ventilated apartments, being rendered perfectly harmless by dilution with the atmosphere,—a security, the credit of which may allowably be claimed by the innocent camphor-bag. It may be satisfactory also for the public to be informed, that this fever has been invariably of a mild character; and it may be an additional satisfaction to know that their security is most essentially promoted by the unceasing exertions of the Fever Institution; which not only affords a ready receptacle for their servants and dependents seized with fever, but is actively engaged in removing the sick from the midst of those whom they might infect, and in cleansing and purifying every successive nest of the contagion that is discovered; thus doubly contributing to stay the progress of the epidemic. May I be allowed to add, as a hint to the benevolent, that its funds are altogether inadequate to the extraordinary exertions which it is now called upon to make for the public safety.

I have the honour to be, Sir,

Your very obedient servant,

T. BATEMAN, M.D.

*Bloomsbury-square; Oct.* 20, 1817.

*To the Editor of the Times.* — Sir,—My situation as Senior Physician to Guy's Hospital renders it incumbent upon me to notice, and to correct, the impression which is likely to be made upon the public mind by certain misstatements lately published respecting the greater or less prevalence of contagious fever in London this year than for several years past. The public attention appears to have been first drawn to this question by a letter from Dr. Brine, of Cavendish-square, purporting to allay the alarm said to prevail respecting the existence of a bad fever in this metropolis, by assuring the community that no such fever existed, and appealing to his professional brethren for a confirmation of this opinion. That letter has been properly answered by Dr. Bateman, whose situation as Senior Physician to the Fever Institution gives him the most extensive means of knowing the state of fever among the poor throughout the metropolis; and who proves, that the number entered upon the books of the Institution, in the present year, amounted to *one half* the collective number for the whole of the last *ten* years; and that this fever prevails chiefly among the poorest, worst fed, and worst clothed persons, and certainly assumes more of the low and typhoid character than has been observed for several years past. Dr. Brine, not being physician either to an hospital or dispensary, can have but few opportunities of seeing such cases; for, if his practice be extensive among the higher ranks, these are seldom the subjects of such fever. But, as the question is thus decided by sound judgment and extensive means of observation, I should have allowed the matter to rest, had I not seen, with the utmost surprise, a letter in your journal, signed T. Warley, and dated from Guy's Hospital, assuring the public that fever was not more prevalent in that, and several other hospitals which he had visited, than on former years. Who is the person so signing his name to a falsehood I know not, as no such person either is, or ever was, a medical officer of Guy's Hospital; nor do I find his name even among the pupils studying there. My own observation on the practice of this Hospital is certainly in favour of Dr. Bateman's statement, viz.—that fever, assuming more of the low and typhoid character, is now more prevalent than for many years past; but most of the cases have done well.

It may not be amiss here to remark, that, unlike the contagion of small-pox, measles, scarlatina, and all others affecting a patient but once during life, and which is always propagated by the peculiar disease alone, the contagion of typhus fever may be generated *de novo* by poverty, depressing passions, filth, bad air, and crowded bed-rooms; as in prisons, hospital-ships, &c.: and (what is of equal, if not greater, importance,) that the generation of this may be prevented, and its spreading avoided, by cleanliness, ventilation, and wholesome food.

I hope that what is here said will dissipate unnecessary alarm among the better ranks on the one hand, and prevent some philanthropic people from running into danger's mouth, by their desire to visit and relieve the poorer classes in person. Their contributions to the Fever Institution will do more good than ten times as much expended in wine and good things for the sick poor at their own homes. Your obedient servant,

JAMES CURRY.

*Bridge-street, Blackfriars; Oct. 26.*

*To the Editor of the Sun.*—Sir,—It gives me much pleasure to find, in the example of other physicians, an apology for addressing the public on the present State of Health in the Metropolis. As early as the year 1809, when the town was particularly healthy, I took some pains to show the importance of providing against such an event as had occurred at the beginning of the present century. There was then no appearance of danger; and, though it might seem to require no force of rhetoric to prove that a fever, originating from the distressed condition of the labouring poor, had ceased with its cause, yet a general opinion prevailed that the fever had ceased, and, consequently, might be exterminated by the exertions of the Fever Society.

We are told, "the fever is doubtless contagious, but chiefly, almost exclusively so, in the close and crowded habitations of the indigent; and the public may be assured the contagion cannot be communicated in the open air or in well ventilated apartments, being rendered perfectly harmless by dilution in the atmosphere; a security, the credit of which may be allowably claimed by the innocent camphor-bag."

To show the looseness of this language, I need only ask any of your readers (the writer, I am sure, has already anticipated my opinion,) whether the contagion of the small-pox, or measles, or scarlet-fever, is thus confined? whether any dilution, which can be ascertained, is a sufficient security? and whether, when those diseases invade a village, they do not spread with more certainty than in the confined air of the metropolis? The answer may be, "all that is asserted is the difference in the degree of contagion." But such is not the case. It is the different manner in which the contagion spreads, and, consequently, in which we are to provide against it: when these two subjects are duly attended to, we shall find that the opinion given by the above writer is dangerous, and his supposed security altogether inadequate.

So far from the contagion not spreading in the open atmosphere, it is certain that paupers brought from jails, or from the dwellings of extreme wretchedness, rendered still more confined by the conscious guilt of some one of the inhabitants, have, by the effluvia from their clothes and bodies, induced a mortal fever on those who only saw them in open apartments. In the Black Assizes (as it was called) in the year 50, when the prisoners at the Old Bailey were well enough to attend their trials, near twenty of the court died from the effects of the effluvia brought by those prisoners; and, so far was that "contagion" (to continue the expression) "rendered perfectly harmless by dilution," that it is well known the greatest sufferers were seated near to an open window.

These unfortunate victims, however, infected none of those by whom they were nursed and visited in their own open apartments. There is, therefore, every physical certainty that the disease may be *received* in the freest atmosphere from a person just arrived from an infectious quarter, yet that it cannot afterwards be *continued* by a clean person in a well ventilated apartment.

The inference from all this is,—1st. That no one can with safety expose himself, under any circumstances, to a mendicant, or prisoner, who inhabits an infectious dwelling, or who arrives from an infectious quarter, with clothes and skin long impregnated with such an atmosphere.

2dly. That no clean person, thus accidentally exposed and infected, if nursed in a well ventilated apartment, is in any respect contagious. It is necessary to be aware of this, because the fever, though principally found, and spreading only in the resorts of the indigent, appears also among those of better condition, from causes which, if you and the public will permit, I shall gladly explain in a future letter. At present, it may be enough to remark that, as there can be no necessity for removing a servant from a well regulated house, there is the greatest impropriety in sending one to the Fever-house. Should that asylum become crowded, there is a greater certainty that the infectious atmosphere will be formed within its walls; and thus the whole intentions of the Establishment be frustrated, and the building prove worse than useless.

I am, Sir, your obedient servant,

*Hatton Garden; Oct. 25.* JOSEPH ADAMS.

*To the Editor of the Morning Chronicle.*—SIR,—Great alarm has been spread in the metropolis by a report of the prevalence of fever. Febrile diseases have certainly predominated, but this alarm has been unnecessarily aggravated by the misapplication of the word *typhus.*

From holding a situation in the House of Recovery, I have been called upon to visit some poor families labouring under fever. I find it entirely restricted to low situations, arising from a want of cleanliness and nutritious diet; but, even in the parts most infected, it has no where (within the limits of my observation) extended to confirmed typhus, but merely assumed the form of simple synochus, or low nervous fever. The inscription on the Fever Institution, importing it to be a House of Recovery from Typhus, has also added to the general alarm, for, from the numerous applications and admissions daily, the mind of the public has been impressed with an idea, that all these applicants are labouring under typhus, when, in fact, this is far from being the case. I am in attendance on several families in the vicinity of the institution, and am induced to make these observations, from having frequently witnessed the fears thus unnecessarily excited. I consider too, there are other causes for the increase of our patients at the House of Recovery, more preponderating than an unusual prevalence of fever. In the first place, the additional publicity of the institution, by its removal from Gray's-inn-lane to the Pancras-road; and, secondly, the consequent increase of its subscribers, including, within the last twelve months, several parishes: these parishes, from the severity of the times, have been overflowing with poor, and, to make room for the distressed, have been glad of an opportunity of removing the sick, by sending us five or six at a time, though not afflicted with confirmed typhus. It must be remembered also, that neither time nor interest is required for the admission of a patient, but merely the signature of a professional man; and an institution so beneficial in itself, and so productive of public good, now once known, will always be resorted to.

1, *Clarendon-square, Somers-town; Oct.* 27, 1817. M. ROWE, Surgeon, &c.

*To the Right Honourable the Lord Mayor, and the other Gentlemen who attended the Meeting at the City-of-London Tavern, on account of the Contagious Fever.*

MY LORD AND GENTLEMEN.—I cannot help wishing, that the present alarm may excite those inquiries which I took so much pains to urge more than eight years ago.* My object was to ascertain, if possible, the different laws by which the various diseases, generally supposed to agree in their contagious properties, are go-

---

* In a tract, entitled, "Inquiry into the Laws of different Epidemic Diseases, with a View to ascertain the Means of Preserving Individuals and Communities from each; 1809."

verned. If they all spread in a similar manner, the same caution is applicable to all; but, if some are governed by climate, others by season, others by local circumstances, and, lastly, if some are contagious in all climates, in all seasons, and under all circumstances of locality, common sense would teach us that different means should be used to protect ourselves and the public from each. The yellow-fever is known only where the heat remains for a certain term at a degree which always arrests the plague. These two diseases have never hitherto been found in the same countries, and both of them, in the countries in which they occur, cease with a certain change of temperature. The means of escaping these, therefore, is to avoid those towns, or those quarters of a town, in which they are known to rage, until the pestilential season ceases. The hospital, poor-house, prison, ship, or camp, fever (as it is very properly called, according to the place in which it originates, but which of late, with fevers from many other causes, has assumed the undefined term of Typhus) originates from various causes in the places which give it name, and spreads by the atmosphere inhaled, whether on the spot or from substances—principally articles of apparel much imbued with it. If a person, by only casual exposure, catches this fever, his apparel will be little imbued, and his own person, however severe the fever may prove, will not be dangerous in a well-ventilated apartment.

This is different from the true contagious. Small-pox, scarlet-fever, measles, and some other diseases exist at all seasons, in all climates, in all temperatures, and in places under all known circumstances; every individual under either of them is contagious, and every one affected by him is contagious also. The purity of the air in other respects, which effectually puts a stop to the hospital-fever, increases the activity of these contagions. The quantity of contagious matter makes no difference in the subsequent disease; persons exposed to the bedside of one who has died of scarlatina, have had the disease as mildly as those who have received it so imperceptibly that the quantity of matter inhaled must have been very inconsiderable. Against these contagions, we know but of one security. The same power which permitted these evils, has so restricted them, that no one is susceptible of them after having once passed through the disease. Without this provision, the multiplication of contagions, which there is reason to believe were unknown to the ancients, would render life not worth acceptance, on account of the constant apprehension we should feel from every stranger, from most articles of commerce; and on account of the necessity of so often abandoning our nearest connexions, or the chance of leaving them to lament our loss in consequence of our piety towards them.

In the limits of a periodical paper, room cannot be expected to expatiate on these subjects. But, I trust the present events will apologise for repeating a small part of what was urged in the tract alluded to, concerning the present most exaggerated reports and consequent alarm.

The fever from poverty, as I shall take the liberty of calling it, was alarmingly prevalent at the close of the late, and the beginning of the present, century, when the distresses of the labouring class was beyond comparison greater than at present. It ceased, however, as their condition mended, and has now returned with returning distress. The proper inquiry is, how we are to protect ourselves from it, and how to exterminate it altogether.

When a poor family is affected with this disease, they should only be visited by the clergy and faculty, or nurses accustomed to the air of sick rooms. The room, or house, should be as much ventilated as possible, and a good fire consequently kept up. It will, for the most part, be better that the sick person and family should remain in their habitation. They have all been exposed to the cause and their cloaths are all imbued to a certain degree. By removing them, therefore, they become dangerous in every place to which they resort, whether they have received the disposition to the fever or not. Before leaving the house, after the recovery of such as are affected by the fever, their cloaths and skin should be washed, as well as every part of the house and furniture.

If a person in better circumstances, or, which is more common, a servant by some intercourse with her sick relations, or their clothes, has caught the fever, there will be no other reason for removing such a subject, excepting the inconvenience from a sick domestic in all but very large establishments.

The next inquiry is, the means of exterminating such a fever altogether. We have seen that an improved condition of the poor is, on a great scale, sufficient for this purpose. But, in a large commercial town, and still more in great manufacturing towns, the fluctuation of employment is always attended with partial, oftentimes unknown, poverty and its attendant evils; and, whenever from any concealed cause, such a fever is excited, it has been shown that there is danger of its extending to an individual residing at a distance from the source of the disease, by being only casually exposed to the air of such dwellings, or to articles imbued with it. As this person will probably be the only sufferer among his own connexions, the cause is not even suspected.

There are two objects, therefore, to be considered, first, to ascertain the spots in which fever prevails; secondly, so to improve the condition of the poor as to lessen, if not entirely to remove, the cause of such fevers.

For the first, some provision has been made by our weekly bills of mortality. These are entirely in the hands of the parish clerks, who receive their information from certain old women called searchers. This office is usually given to some poor creatures whom the parish must otherwise support. Their business is to examine whether there are any marks of violence on the deceased; their fee is a shilling each, and, in many places, they expect a dram also. It is needless to dwell on the uncertainty of reports from such sources. St. Pancras and Mary-le-bone are not included in the bills of mortality; yet, it has been ascertained, that the latter contains more souls than all Bristol, including Clifton and its environs. The regularity with which its concerns are managed, induced me to hope that the respectable trustees would adopt a plan proposed in an "Appendix" for ascertaining the state of health and mortality in that extensive district, or any other mode which they might find more suitable, and which, when matured, might serve as an example to the whole empire.

Not succeeding with any of the gentlemen to whom I applied, my next attempt was with the corporation of London, but the parish clerks were already in possession of the office.—My application to the officers of the Company of Parish Clerks ended as might have been expected. But, I cannot see that their function precludes the corporation from every attempt at ascertaining the health of the city; I have, therefore, some hopes in the enquiries which the present alarm may excite.

My other attempt has been, I trust, most completely successful, and, had it taken place in the flourishing year of 1809, when it was first published, would have greatly, if not entirely, prevented the present distress, excepting in those discharged from the army and navy. Nor is it too much to believe, that it might have prevented those disorders which have so disgraced the metropolis and some of our manufacturing towns.

This was no other than the present Saving Banks*, which I have had the happiness to find almost universally received, and, most of all, by some who discouraged me the most.

The above will, I hope, be sufficient to show, that, however no-

---

* To show that my objects were exactly similar to those now universally approved, and that I had the public tranquillity in view, though the public health was all that my professional duty authorised me to bring forward, I shall transcribe the following short extracts from my plan, published nine years ago.

"The only plan, to be permanently useful, must be completely within their comprehension, liable to no uncertainties, and by always keeping within their view the true value of money, induce economy in the management and diligence in the acquisition of it.

"May not a bank be established ready to receive the smallest weekly contribution?"—Every half-year the balance should be struck, and interest for six months added, &c.

"The Bank of England, it is true, will derive little or no profit and a certain expence, but, in my opinion, that grand establishment will derive ample advantage from the general interest all the Londoners will feel in supporting or submitting to their charter. It is not probable, that the bank should ever feel any other danger than this jealousy; but, should such a moment occur, no better security can be desired than the interest which would be felt in its favour by so numerous a body of that class of citizens which compose their new customers."

*** In justice to such bank directors as I applied to, it should be added, that their charter precluded them from paying interest for money lodged in their hands: and, in justice to Mr. Rose, that, by an Act of Parliament, he produced the necessary security, and the other provisions required in my appendix.

vel the question agitated might be to gentlemen whose pursuits have been different, it had occupied my mind long enough to acquire some maturity, and this, I trust, will prove a sufficient apology for any unexpected intrusion, on an occasion of promoting the best worldly interest of the community. I have the honour to be,

My Lord and Gentlemen,

Your faithful humble servant,

JOSEPH ADAMS.

*Hatton-garden;*

*Nov.* 23, 1817.

We shall conclude this article with a few remarks on language, to which we are always earnestly craving the attention of our readers. The terms we wish them particularly to keep in view are *Endemic* and *Epidemic*, *Infection* and *Contagion*. Of the two first we should say with Scaliger, *nam*, Το ενδεμιον, *endemicum est, quod* IN *populo*, επιδημιον *vero quod vagatur* PER *populum, grassatur et populatur*. Ενδεμος *enim* νοσος *potest esse, citra aeris vitium*. Επιδεμια *non potest*.

Nothing appears more simple than such a distinction; yet we find the accurate Vanswieten mistaking its meaning whilst he attempts to illustrate it:—"*Si ergo loci natura et situ, morbi oriantur, illorum causa perennis manet semperque adsunt et dicuntur* ENDEMII: *si autem quodam tempore tantum regionem pervadunt* VOCANTUR EPIDEMICI." Here it is evident that this industrious writer, as soon as he leaves his copy, becomes confused. Agues may be said to arise *loci natura*, yet they are not always present. The true endemic diseases, if not the effect of climate, are probably, for the most part, the consequences of some hereditary predisposition, strengthened by repeated intermarriages in confined circles. Of this nature the goitres are suspected to be; an opinion strongly confirmed by Governor Raffles, in his History of Java, vol. i. p. 60.

"Here, as on Sumatra, there are certain mountainous districts, in which the people are subject to those large wens in the throat, termed in Europe *goitres*. The cause is generally ascribed by the natives to the quality of the water; but there seems good ground for concluding that it is rather to be traced to the atmosphere. In proof of this it may be mentioned, that there is a village near the foot of the Teng'gar mountain, in the eastern part of the island, where every family is afflicted by this malady; while in another village, situated at a *greater elevation*, and through which the stream descends which serves for the use of both, there exists no such deformity.—These wens are considered hereditary in some families, and seem thus independent of situation. A branch of the family of the present *Adipáti* of Bándung is subject to them, and it is remarkable that they prevail chiefly among the women in that family."

The hereditary property of this disease is the only one in which all writers agree. In the Alps and in Derbyshire an elevated situation is supposed necessary; but Dr. Barton in America, and Governor Raffles in Java, found, only in valleys, goitres. The disease therefore, being unconnected with air or soil, is strictly *endemic*.

Those diseases which arise from *loci natura et situ*,—that is, from the soil,—were by some very properly called ἐπιχώριοι; and, as they are not constantly present, they may be called epidemic when they occur. Camp-fevers and all the contagious, as often as they spread generally, may also, in common language, be called epidemic. But it would be more correct to confine this term to those diseases which seem to travel from country to country, without requiring any particular soil or any source of infection. Of this kind, the most familiar to this nation and age is the Influenza, as it is generally called. The sweating sickness was probably the same in this respect; and it is suspected by some that the plague is governed by no other laws, excepting that its effects are more partial, and that a certain temperature will always arrest it.

If these two terms, Endemic and Epidemic, are thus confined, Infection may be restricted to that kind of fever which is generated in camps, hospitals, poor-houses, &c. in which places the air may be *infected* or *stained*, and the disease which follows is in some degree modified by the quantity of impurity, or staining matter, contained in a given portion of the air; so that a person only suddenly exposed, and afterwards nursed in a well ventilated apartment, will not impart sufficient impurity to the air to injure any one.

This is different from the small-pox and other *contagions*, as they may very properly be called; because the slightest *contact* between a diseased and a healthy subject, or the contact of the air, however diluted, if sufficient to induce disease, may induce it in as violent a form as if the healthy subject had been in actual contact with the deceased, or had received the atmosphere loaded in the highest degree with these deleterious particles. If these distinctions are admitted—

*Endemic* diseases are hereditary, and become more fixed in proportion as a community is more secluded from the rest of the world. They are, therefore, only to be lessened by a more general intercourse.

*Epidemic* depend on no other cause than some constitution of the atmosphere with which we are unacquainted, and, as has been observed, can only be avoided by keeping clear of the places known to be under the influence of such a cause.

*Epichorii* depend on the nature of the soil at certain seasons, during which such places are to be avoided.

*Infections* depend on a confined air among sick, labouring under any kind of disease. The means of escape is to keep free from such places, and from whatever is embued with the air of them.

*Contagions*, such as small-pox, scarlatina, &c. depend on their specific contagion, with the origin of which we are unacquainted, but which at present we can only trace from those affected with the disease itself. We know of no safety from these, but in having passed through the disease.

We submit these definitions to our learned readers, and shall be thankful for their corrections before we enter on our long-promised remarks on Contagion.

